# Green Nanocomposites Based on Thermoplastic Starch: A Review

**DOI:** 10.3390/polym13193227

**Published:** 2021-09-23

**Authors:** Katherine E. Rivadeneira-Velasco, Christian A. Utreras-Silva, Antonio Díaz-Barrios, Alicia E. Sommer-Márquez, Juan P. Tafur, Rose M. Michell

**Affiliations:** School of Chemical Sciences & Engineering, Yachay Tech University, Urcuquí 100119, Ecuador; katherine.rivadeneira@yachaytech.edu.ec (K.E.R.-V.); christian.utreras@yachaytech.edu.ec (C.A.U.-S.); adiaz@yachaytech.edu.ec (A.D.-B.); asommer@yachaytech.edu.ec (A.E.S.-M.); jtafur@yachaytech.edu.ec (J.P.T.)

**Keywords:** thermoplastic starch, nanocomposites, biopolymers, properties, packaging, nanofiller

## Abstract

The development of bio-based materials has been a consequence of the environmental awareness generated over time. The versatility of native starch is a promising starting point for manufacturing environmentally friendly materials. This work aims to compile information on the advancements in research on thermoplastic starch (TPS) nanocomposites after the addition of mainly these four nanofillers: natural montmorillonite (MMT), organically modified montmorillonite (O-MMT), cellulose nanocrystals (CNC), and cellulose nanofibers (CNF). The analyzed properties of nanocomposites were mechanical, barrier, optical, and degradability. The most important results were that as the nanofiller increases, the TPS modulus and strength increase; however, the elongation decreases. Furthermore, the barrier properties indicate that that the incorporation of nanofillers confers superior hydrophobicity. However, the optical properties (transparency and luminosity) are mostly reduced, and the color variation is more evident with the addition of these fillers. The biodegradability rate increases with these nanocompounds, as demonstrated by the study of the method of burial in the soil. The results of this compilation show that the compatibility, proper dispersion, and distribution of nanofiller through the TPS matrix are critical factors in overcoming the limitations of starch when extending the applications of these biomaterials. TPS nanocomposites are materials with great potential for improvement. Exploring new sources of starch and natural nano-reinforcement could lead to a genuinely eco-friendly material that can replace traditional polymers in applications such as packaging.

## 1. Introduction

**Figure 1:**
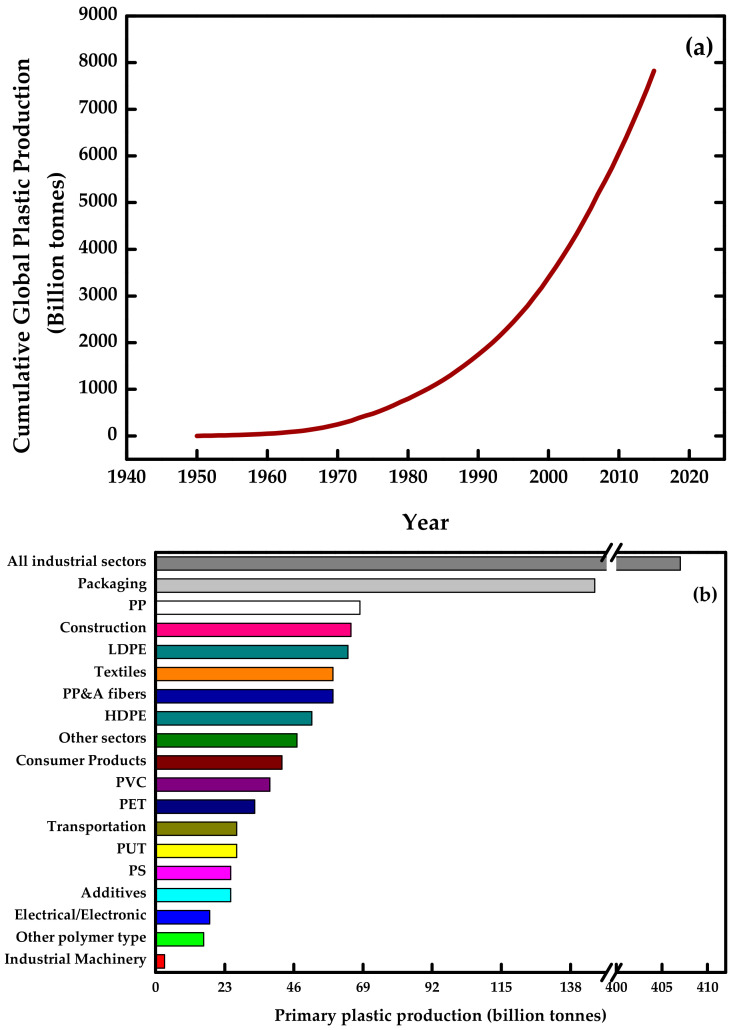
Plastic production: (**a**) global cumulative from 1950 to 2015, (**b**) primary in different entities at 2015. Prepared from data in [1].

Plastic production has been increasing over time (Figure 1a) [1]. It is currently one of the most used materials worldwide due to its excellent flexibility, durability, and resistance. This type of material also allows the modeling of various products that can be applied in different fields of human activities (e.g., electronics, medical supplies, building, and packaging, among others) [2]. However, plastic represents a potential pollutant for the planet because of poor recycling methods and the limited capacity of storage facilities. As a result, the plastic accumulation is greater than the decomposition rate of this material in landfills or the environment [1].

According to the UN Environment Programme (UNEP) [3], from the 1950s to the present, more than 8.3 billion tonnes of plastic have been produced, of which around 60% has ended up in the natural environment and a part of them in the oceans (4 to 12 million metric tonnes [1]), which tend to produce substantial damage to marine species. Generally, carbon-based products are from non-renewable sources. Another problem with plastic is that many of the additives used in its manufacture are not biodegradable, and in some cases, they can be toxic [4]. Furthermore, waste disposal and the depletion of fossil sources are the major drawbacks of maintaining non-biodegradable single-use products [5]. Therefore, the replacement of oil-based products with bio-based products is the driving force behind these initiatives.

Numerous studies have been conducted in search of sustainable and ecological alternatives to deal with the excessive production of plastic derived from fossil hydrocarbons [6,7,8,9,10]. The main objective is to find low-cost and biodegradable materials with good mechanical properties and high quality [11]. One of the alternatives is biopolymers manufactured from biomass or organic waste, which come from natural sources because they are abundantly available and inexpensive. Consequently, they are economically viable compared to synthetic oil-based products [2,12]. Thermoplastic starch (TPS) is becoming an interesting option to achieve this goal.

TPS is a biopolymer prepared from native starch after its granular structure transformation using a plasticizer (water, glycerol, and sorbitol, among others) [13]. Native starch is abundant in nature and takes advantage of these low-cost resources obtained from agroindustrial waste. TPS alone is not suitable as a work material due to its poor performance. To enhance the final product, blending with other polymers and incorporating additives into the matrix is mandatory. The most common blends of TPS/polymer are prepared with poly(lactic acid) (PLA) [14,15,16,17,18], polyethylene varieties [19,20,21,22,23,24,25,26], poly(ethylene-co-vinyl alcohol) (EVA) [27,28], polycaprolactone [29], poly(vinyl alcohol) (PVA) [30], polyester [31], and polypropylene [32]. Additionally, a promising additive employed to reinforce the TPS is nanofillers to obtain nanocomposites-type materials. Consequently, they have been widely developed because of the synergistic merging of nanofillers in the polymer matrix of TPS.

The nanofillers that can be used for TPS reinforcement could be extracted from natural sources: cellulose [33,34,35,36,37], native starch [38,39,40,41,42], clays [43,44,45,46,47,48], and chitosan [49]. The most used nanofillers are natural montmorillonite (MMT), organically modified montmorillonite (O-MMT), cellulose nanocrystals (CNC), and cellulose nanofibers (CNF). Nanofillers (nanofibers, nanocrystals, and nanoparticles) obtained from polysaccharide materials are among the best options because they have similar chemical structures to TPS and are easy to extract.

Furthermore, green nanocomposites based on TPS help to reduce the environmental impact caused by oil-based products, and they can have several applications in different fields, such as the food industry [47,50,51], packaging (one of the largest plastics markets, as shown in Figure 1b) [1,52,53], drug delivery [54], biosensors, and electronic shielding [55], and tissue engineering [56].

Some reviews have been organized around different approaches to or related to TPS. Processing techniques for TPS with emphasis on the importance of plasticizer [9] and TPS nanocomposites (highlighting the novel water-assisted technique) [7] have been reviewed. In the same way, there are some others that present the characterization of TPS-based materials, including polyblends (e.g., PVA/starch) [4] and composites [6,8,10,57]. Recently, the use of TPS as a food packaging material has been studied [9,58]. However, no reviews exclusively focus on completely natural-based materials reinforcement for TPS and its potential packaging application.

For the above reason, this study will conduct a critical literature review on TPS-based nanocomposites and how these materials could substitute for conventional packaging plastics. It will present an overview of natural nanofillers (MMT, O- MMT, CNC, and CNF) and how they improve the TPS properties, mainly mechanical, barrier, and optical, as well as the degradability. Finally, this review could be a starting point for future research on environmentally friendly packaging materials based on TPS.

## 2. Thermoplastic Starch

**Figure 2:**
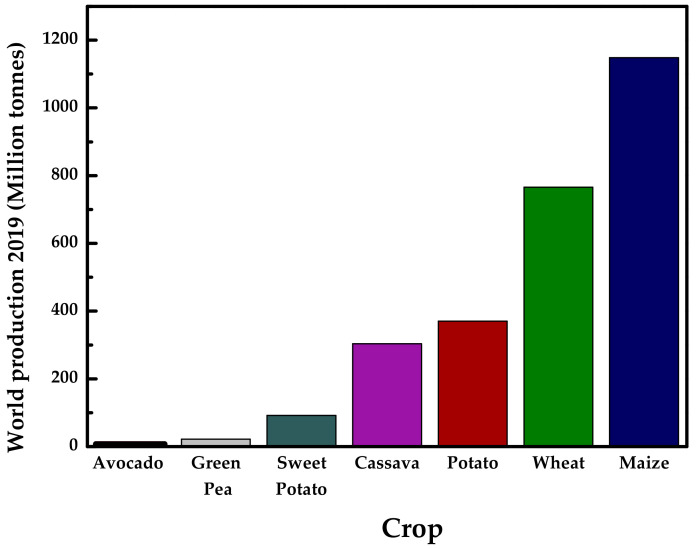
World production of main starch crops in 2019 (million tonnes). Prepared from data in [94].

Native starch is a renewable natural resource, which can be extracted from different parts of the plants, such as seeds, fruits, leaves, tubers, and roots. It may come from a variety of vegetables and greens [56], including potato [34,46,59,60,61,62,63,64], cassava [65,66,67,68,69,70,71], maize [28,38,72,73,74,75,76], wheat [58], pea [5,45,77,78,79,80], tapioca [2,81,82], corn [33,35,36,43,47,48,83,84,85,86,87,88,89], pomegranate [90], sweet potato [53], and avocado [91,92,93]. Figure 2 shows the world production of some of the mentioned sources, which have been increasing over time [94], looking forward to promoting the usage of their residues.

Starch granules are mainly composed of amylose (linear component) and amylopectin (branched component). The functionality and applicability of starch are given by its two high-molecular-weight components. They vary depending on the natural source: 20–30% of amylose and 70–80% of amylopectin [8]. The amylose/amylopectin ratio and the non-starch components (such as lipids, proteins, and phosphate groups) from the natural source will determine the properties of the starch [95]. In general, the functional properties of starch are associated to its water absorption, which is related to the amylose amylopectin ratio [96].

Starch has a semicrystalline structure (the crystallinity degree varies between 15% and 45% [97]), in which the amorphous region comprises amylose and long amylopectin chains, while the crystalline region comprises short amylopectin chains [98]. If there is a higher content of amylose, it directly affects the organization of the crystalline lamellae in the granules as well as the amylopectin packaging degree [96]. Consequently, the ratio of amylose and amylopectin affects the granular shape and morphology of starch.

**Figure 3:**
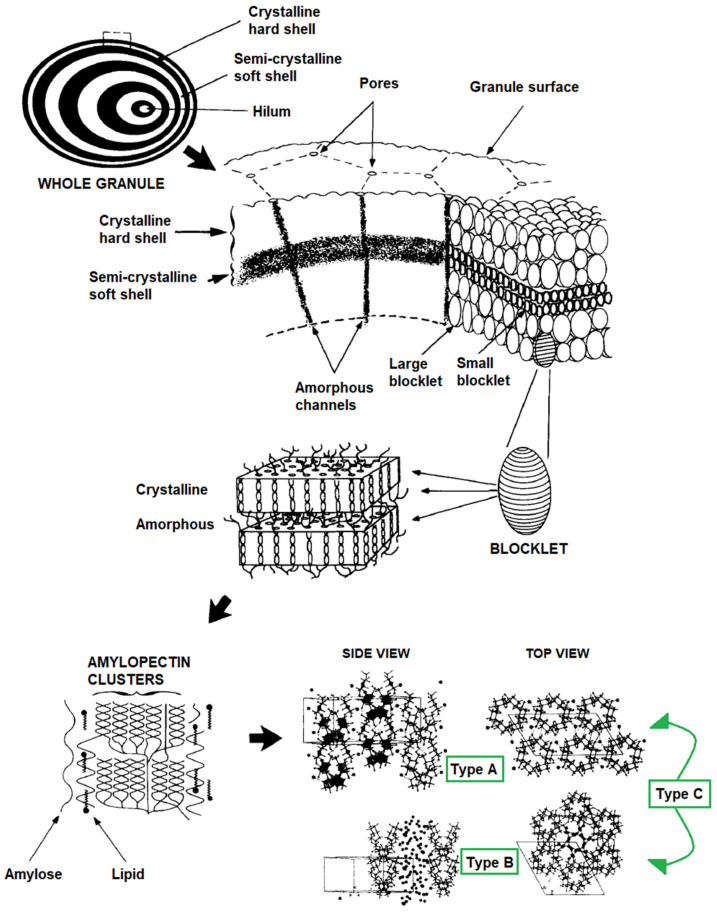
Crystallographic description of the native starch granule. Reproduced with permission from Carbohydr. Polym., 32 (3–4), Gallant et al., Microscopy of Starch: Evidence of a New Level of Granule Organization, 177–191, 1997 [99].

The crystalline structure of starch shows three polymorphisms; these are classified into types A, B, and C (see Figure 3). Type A is formed from orthogonally packed double helices with a firm inclusion of structural water, type B presents the double helices in a wider orthogonal packing with 36 water molecules per unit cell (some of them are located in the channels formed by the hexagonal packing), and type C exhibits both polymorphisms, A and B [99].

**Figure 4:**
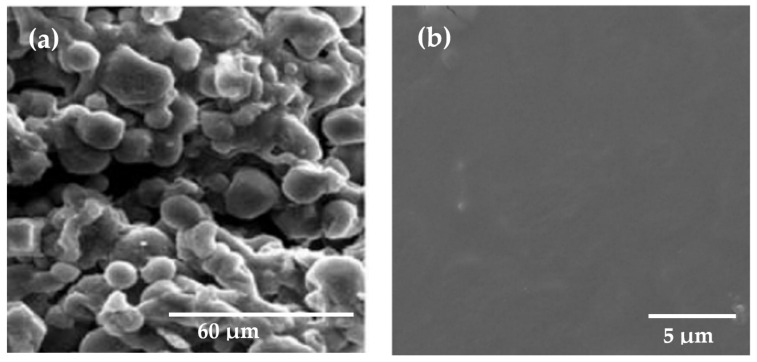
(**a**) SEM representation of native granular starch. Reproduced with permission from Polym. Environ., *17*, Ren et al., Study on biodegradable starch/OMMT nanocomposites for packaging applications, 203–207, 2009 [53]. (**b**) SEM representation of thermoplastic starch. Reproduced with permission from Fibers, *6*, Asrofi et al., Mechanical properties of a water hyacinth nanofiber cellulose-reinforced thermoplastic starch bionanocomposite: Effect of ultrasonic vibration during processing, 1–9, 2018 [101].

One of the principal industrial applications of starch is plastics when transformed into TPS. It has been developed under specific heat, pressure, and moisture conditions to overcome the impossibility of processing the granular starch using traditional plastic methods. In Figure 4, it can be seen that native starch is discontinuous, and the granules are explicitly shown, while TPS looks like a homogeneous surface with almost no apparent irregularities [100].

TPS preparation differs based on starch source and final product usage, but it may follow the general process below. Materials include extracted starch from natural sources and plasticizers. Assuming that it is necessary to increase the properties, then the use of additives is mandatory. TPS is prepared using the extracted native starch, adding the plasticizer, and taking it to a system equipped with a mixer. Typically, a two-step procedure is followed: the premix is made from a native starch/plasticizer (proportions vary depending on the goal) and held as long as it takes (plasticizer-dependent) to swell the granular starch molecules. The swollen mixture is transferred to the mixer at a certain roller speed to induce gelatinization until the gelatinization temperature is reached. Conditions must be monitored during the process. The final mixture needs to be cooled down and pelletized to be subsequently blended with the additives, improving the properties of the final product [102].

**Figure 5:**
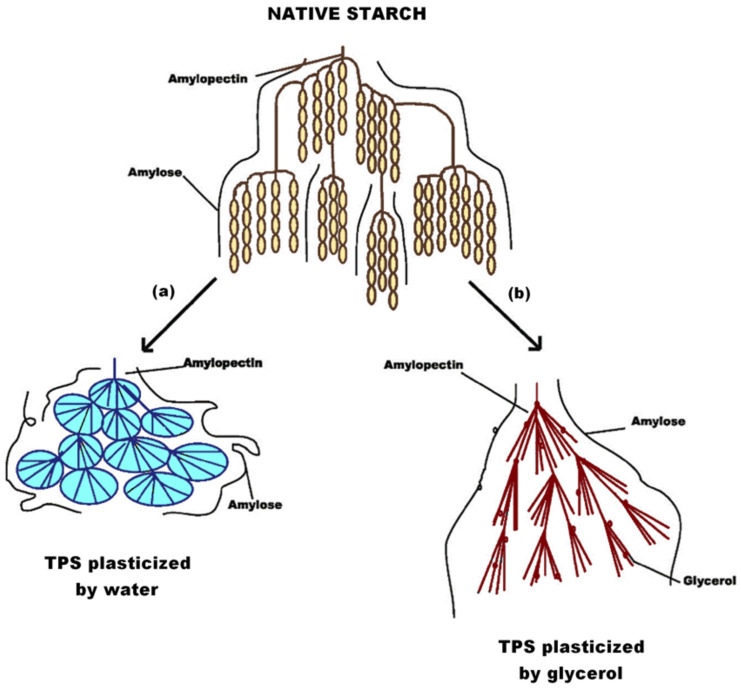
Microstructural change in native starch processing to TPS plasticized with (**a**) water and (**b**) glycerol. Reproduced with permission from *J. Food Process Eng.*, *40*, Khan et al., Thermoplastic Starch: A Possible Biodegradable Food Packaging Material—A Review, 2017 [9].

The role of a plasticizer is critical for achieving the desired product characteristics. It causes microstructural changes in native starch, as can be seen in Figure 5. The molecular size of the plasticizer must be smaller than starch to diffuse within the intermolecular spaces of the polymer and start the interaction [9]. The hydroxy groups of the most common plasticizers (water, glycerol, or sorbitol) make them compatible with starch, generating an adequate interaction [56]. These alter the initial crystallographic structure by breaking the hydrogen bonds that join the macromolecules, with partial depolymerization of the starch structure, allowing the amylose and amylopectin chains to flow with the temperature increase and making the starch thermoplastic [103].

**Figure 6:**
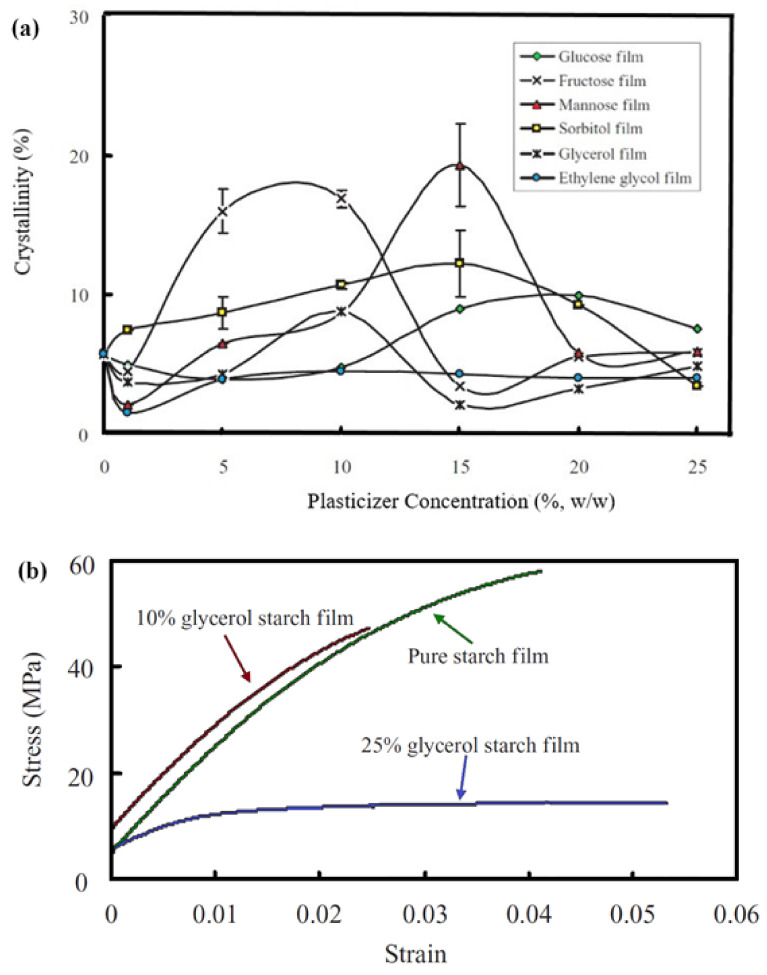
(**a**) Crystallinity dependence of different plasticizers (monosaccharides and polyols) at concentrations from 0 to 25 wt %. (**b**) Tensile test results for starch films varying glycerol concentrations (0, 10, and 25 wt %). Reproduced with permission from *J. Food Sci.*, *75* (*1*), Zhang and Han, Crystallization of high-amylose starch by the addition of plasticizers at low and intermediate concentrations, 2010 [104].

The most common plasticizer for TPS preparation is glycerol due to its properties, such as polarity, hydrophilicity, compatibility, and boiling point lower than the gelatinization temperature. These properties facilitate the gelatinization process by adding flexibility, which implies reducing the viscosity of the molten material [56]. The glass transition temperature, the degree of crystallinity, the amylose content, the type and amount of plasticizer, and the storage conditions are the main parameters involved in the mechanical properties of the obtained TPS materials [8]. Zhang and Han [61,104] studied the properties of pea starch with various plasticizers (monosaccharides and polyols) proportions from 0 to 25% and after 14 days of storage at 50% of relative humidity (RH). Films were prepared by aqueous starch dispersion, keeping them at the boiling point of the starch dispersion during the process. In the case of glycerol (Figure 6) as a plasticizer, it shows an uncommon anti-plasticizing effect on pea starch at low concentrations (less than 10%), while at higher concentrations (around 25%), plasticity was improved and fracture stress was reduced. This phenomenon affects the mechanical properties of starch because at low concentrations (less than 10%), the starch crystals tend to act as physical crosslinking points and produce internal stress, leading to a faster rupture. Based on this example, it is suggested that an optimal amount of plasticizer should be considered to obtain the desired properties of TPS, bearing in mind the nature of starch and/or plasticizer.

In order to promote the microstructural changes on starch, it is necessary to apply temperature, pressures, and shear, typically by extruders or internal mixers. The target temperature of these types of equipment (around 140 and 160 °C) [105] is below the starch decomposition point (230 °C) [103], which implies reducing the glass transition and melting temperatures. The most common methods for TPS transformation are extrusion [106], compression molding [107], and film casting [108]. Injection is not suitable due to the high viscosity and low flow properties of the material. Extrusion is used for film packaging materials, mainly when the twin-screw configuration is used, since it allows adequate feeding and temperature control with a widespread shear. Compression molding is widely used for foam packaging, which involves the gelatinization of starch and lubricating additives to prevent the mold from sticking. Film casting is used to obtain thin sheets approximately 0.02–0.1 mm thick [8].

After transformation, the general characterization of TPS nanocomposites is carried out by X-Ray Diffraction (XRD), Scanning Electron Microscopy (SEM), Thermogravimetric Analysis (TGA), Differential Scanning Calorimetry (DSC), and Atomic Force Microscopy (AFM), among other techniques [102]. Then, the mechanical characterization is generally based on the International Organization for Standardization (ISO) [32,49] or American Society for Testing and Materials (ASTM) [48,62,70,109] and conducted in special machinery for this purpose. With the use of these standards, the performance of the material can be adequately assessed. Using standardized specimens, the most common tests to report mechanical properties are tensile, compression, and flexion [110].

Some of the weaknesses of TPS are the low mechanical, barrier, rheological, and thermal-resistant properties. To improve the performance of TPS, a transformation of this material into nanocomposites is a well-known alternative. These significant drawbacks lead to finding the best options for co-components and nanofillers for the TPS matrix [12]. These nanocomposites are currently applied in different fields because of their properties such as tissue engineering [54], drug delivery systems [54], and packaging [47,111].

## 3. Nanocomposites: General Trend in the Main Properties

The primary purpose of employing fillers is to enhance the properties of TPS. The most common nanofillers found in the literature are CNF, CNC, MMT, and O-MMT. The highest impact of adding nanocompounds to the TPS matrix is improving mechanical, barrier, optical properties, and degradability performance.

**Figure 7:**
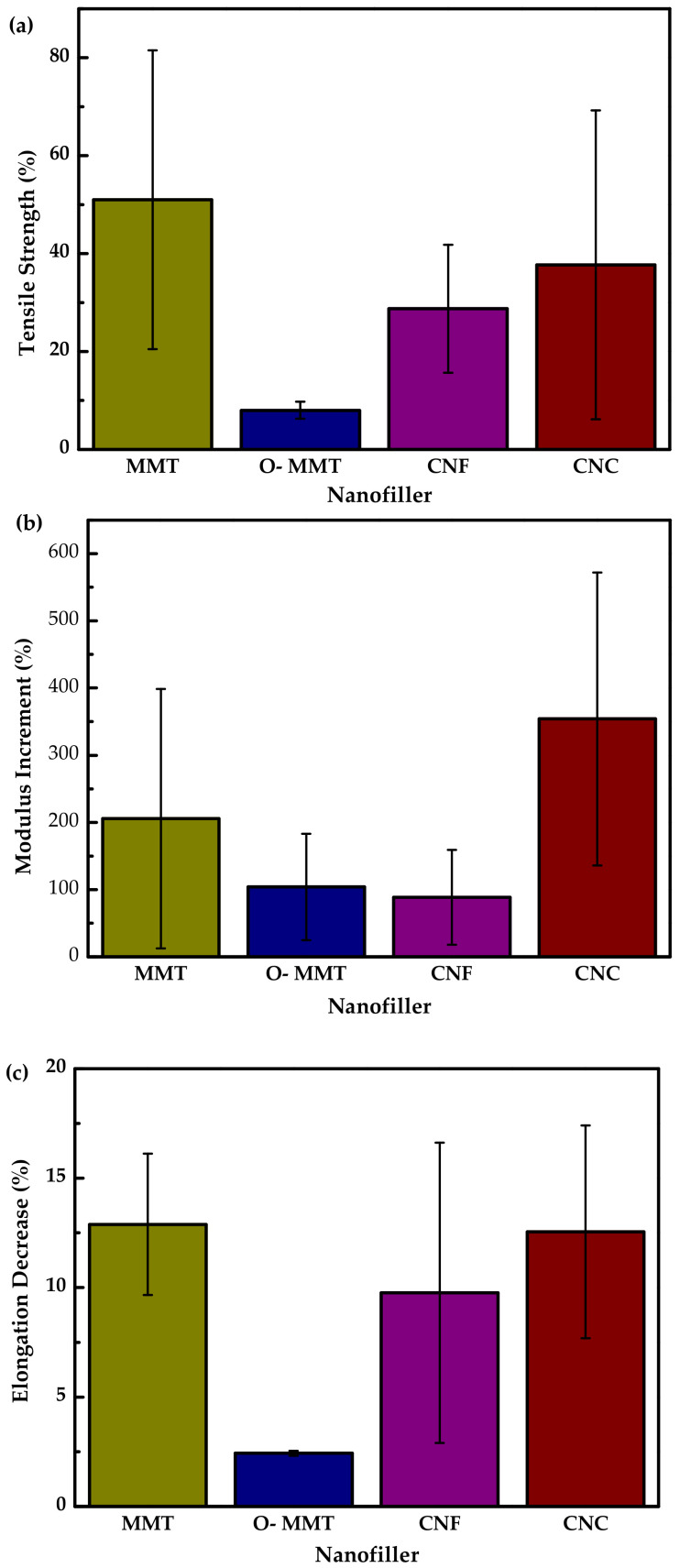
Mechanical properties for the main nanocompounds (MMT, O-MMT, CNF, CNC): (**a**) tensile strength, (**b**) modulus increment, (**c**) elongation decrease. Prepared from data (fixed at 5% of nanofiller) in [20,24,25,26,30,58,66,67,68,74,87,89,90,106,109].

Figure 7 shows a reported mechanical properties compilation of these nanocomposites. The presented values are calculated employing Equation (1) for comparative purposes.

(1) $$\Psi_{\pm }=\left(\frac{\Psi_{\mathrm{filler}}}{\Psi_{\mathrm{blank}}}-1\right)\times 100\%$$

where Ψ_±_ is the reported parameter, Ψ_filler_ is the value of the parameter at a specific filler concentration, and Ψ_blank_ is the value of the parameter for neutral TPS.

**Figure 8:**
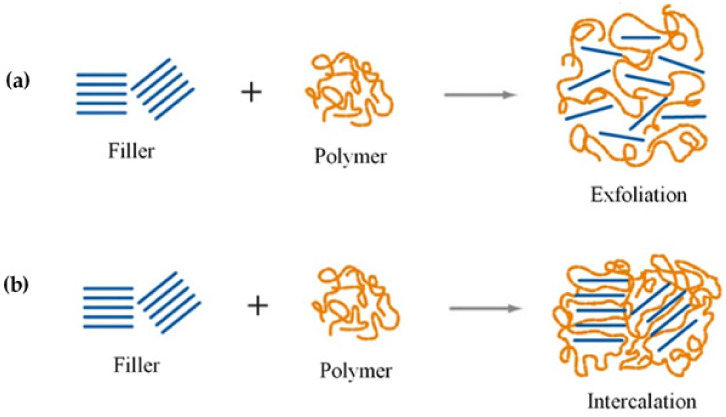
Representation of filler (**a**) exfoliation and (**b**) intercalation within the polymer matrix. Reproduced with permission from *Mater. Sci. Eng. R Reports*, *28*, Alexandre and Dubois, Polymer-layered silicate nanocomposites: Preparation, properties and uses of a new class of materials, 1–63, 2000 [112].

The graphs show the relative increase for strength (Figure 7a) and modulus (Figure 7b) and the decrease in elongation (Figure 7c) for the different nanofillers. It can be deduced that CNC is the one that improves the modulus in a greater way, while MMT shows the highest value of the increase in tensile strength but also the most significant elongation decrease. These results agree with the common trend in the mechanical properties after adding a nanofiller in the TPS matrix, which increases the modulus and tensile strength while reducing elongation. This behavior is related to the exfoliation of the filler (Figure 8). It means that more exfoliation favors modulus and tensile strength, while less exfoliation favors elongation. This fact could help achieve high performance for future applications of this type of material.

**Figure 9:**
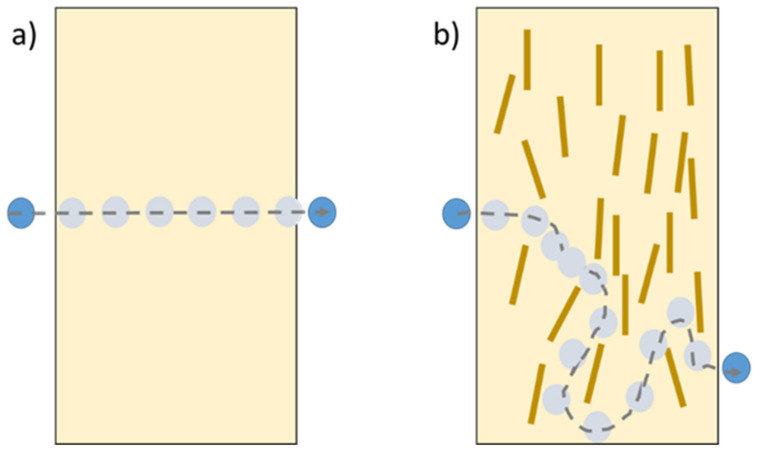
Schematic representation of molecular diffusion through (**a**) TPS and (**b**) TPS nanocomposite.

These nanofillers also improve the barrier properties, such as water vapor permeability (WVP), oxygen permeability (OP), and aroma permeability (AP). They are essential parameters to predict the shelf life of the material on the packaging. However, the most studied barrier property is WVP. In general, bio-based materials exhibit poor WVP [8], so nanocompounds are added to overcome this inconvenience. These nanofillers also prevent the pass of fluids (water, oxygen, and/or aroma) through the film, avoiding the affectation of a marketed product as represented in Figure 9 (the nanofillers cause a torturous path for the molecules).

**Figure 10:**
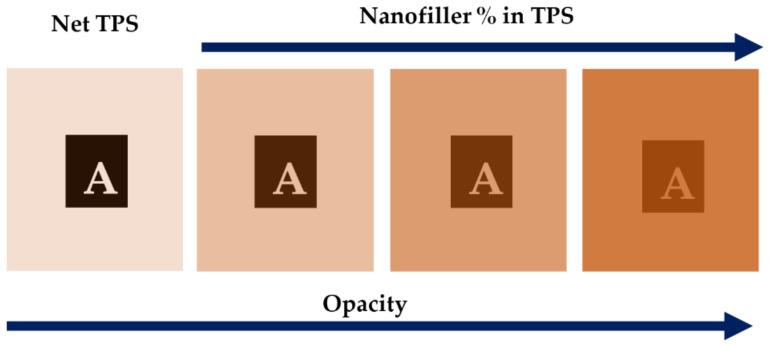
Schematic representation of opacity from net TPS to an increasing percentage of nanofiller in TPS nanocomposite.

The nanofillers modify the aesthetic of the final product, and it is related to optical properties. For example, the transparency of nanocomposites is assessed by transmittance in UV-Vis analysis. When the specific particle size is exceeded (40 nm), the opacity is progressively increased. The Beer–Lambert law can be applied to quantify the loss of clarity [33,44]. In addition, optical transparency can be attributed to light dispersion due to the size of particles that affect the transmittance of light [106]. Bigger particles produce a blockage of visible light, leading to material opacity [22], compared to net TPS that is highly transparent with 90% transmittance at 600 nm [33]. This trend can be represented in Figure 10; the increment of nanofiller percentage in the nanocomposite produces an increase in opacity.

**Figure 11:**
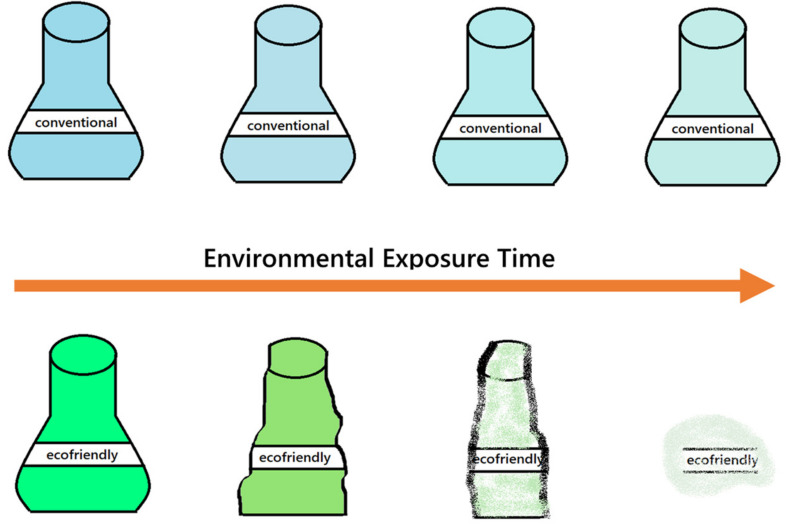
Schematic representation of environmental exposure of a conventional and green (ecofriendly) container with the increase on the environmental exposure time.

Materials based on TPS meet the degradation standard, which means that this material can be converted into biomass, carbon dioxide, and water by the action of biological enzymes in a given environmental exposure time [113]. It should not be confused with the mineralization process because it includes one more end product, methane. This process may never reach 100%, because a part of the polymer will be incorporated into microbial biomass. The polymer degradation path will be determined by environmental conditions (aerobic or anaerobic) [114]. There are many ways in which this property is evaluated for materials, including gravimetric weight changes, morphological changes, mechanical characteristics, and carbon dioxide emission [115]. Figure 11 represents a schematic degradation process for conventional polymers versus eco-friendly/green materials.

## 4. Cellulose Nanofibers (CNF)

Cellulose and starch are the two most abundant polysaccharides in nature; due to their similarity in their structures, there are expected to be no compatibility problems when preparing blends with these components [37]. In this way, the cellulose nanofibers (CNF) could be extracted from various parts of plants, such as pulp, bagasse, husk, and leaves. Table 1 shows the different sources of starch and CNF. The methods for extracting the fibers can be chemical (e.g., acid hydrolysis) and/or mechanical (e.g., high-intensity ultrasonication, high-pressure refiner, grinder treatment, cryocrushing, or electrospinning) [77]. The main advantages of CNF include that they are highly available, easy to degrade, and recyclable.

**Figure 12:**
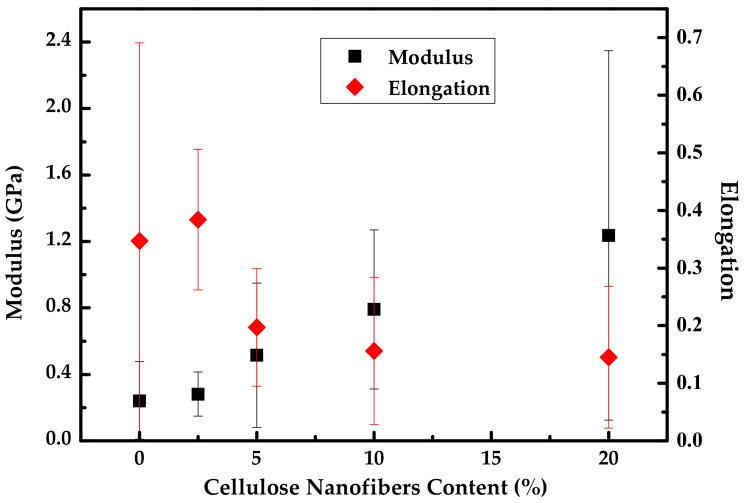
Modulus and elongation trend as CNF content increases in TPS matrix. Prepared from data in [62].

According to the compiled information, CNF is added to the TPS matrix in 0.1 to 20 wt %, showing that in most studies, the presence of CNF increases the stiffness (improvement in Young’s modulus and a slight to moderate decrease in the elongation at break) of the TPS. The corresponding changes are attributed to an adequate filler dispersion in the matrix. Furthermore, the formation of a rigid hydrogen bonds network between the matrix and filler confers adequate stress transfer. This network also implies that an excess of this filler will produce agglomeration, which will lead to poor mechanical properties (Figure 12) [124]. However, Teixeira et al. [70] reported unusual behavior in cassava starch-based TPS and cassava bagasse CNF composites. The main difference in the mechanical properties was the increase in the elongation at break, which was originated by a plasticizing effect of sugars present in the nanofiber suspension through the mixing process, developing a hydrolysis degradation of starch during acid extraction [118].

**Figure 13:**
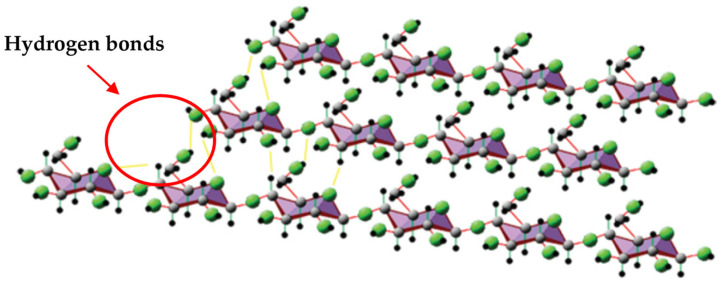
Hydrogen bonds interaction between β-glucose molecules in a segment of cellulose. Reproduced with permission from *Compos. from Renew. Sustain. Mater.*, Pérez-Pacheco et al., Thermoplastic Starch (TPS)–Cellulosic Fibers Composites: Mechanical Properties and Water Vapor Barrier: A Review, 2016 [8].

As was previously discussed, TPS has poor barrier properties; then, with the aid of CNF and its nanometric size, a positive impact on the barrier properties can be observed. A reduction in WVP values is evidenced by promoting a hydrogen bond network between filler and starch chains (Figure 13). This network causes a restriction of diffusion through starch films. Cellulose confers a hydrophilic character to TPS films because of its high crystallinity and compact microfibrillar arrangement [8].

**Figure 14:**
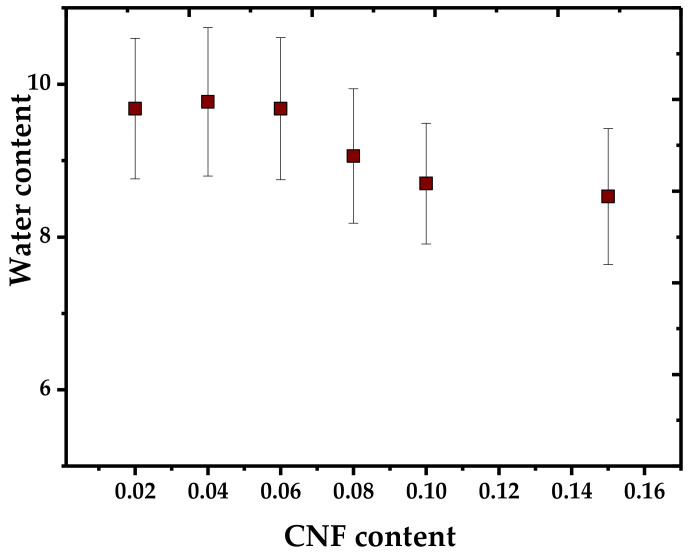
Water uptake trend in respect to CNF content in TPS matrix. Prepared from data in [33].

For example, Fourati et al. [33] studied WVP in nanocomposites based on corn TPS with glycerol (30 wt %) and CNF-oxidized eucalyptus pulp (2–15 wt %). As shown in Figure 14, the expected behavior is obtained; an increase in CNF in the TPS matrix improves the barrier properties, causing lower water uptake.

**Figure 15:**
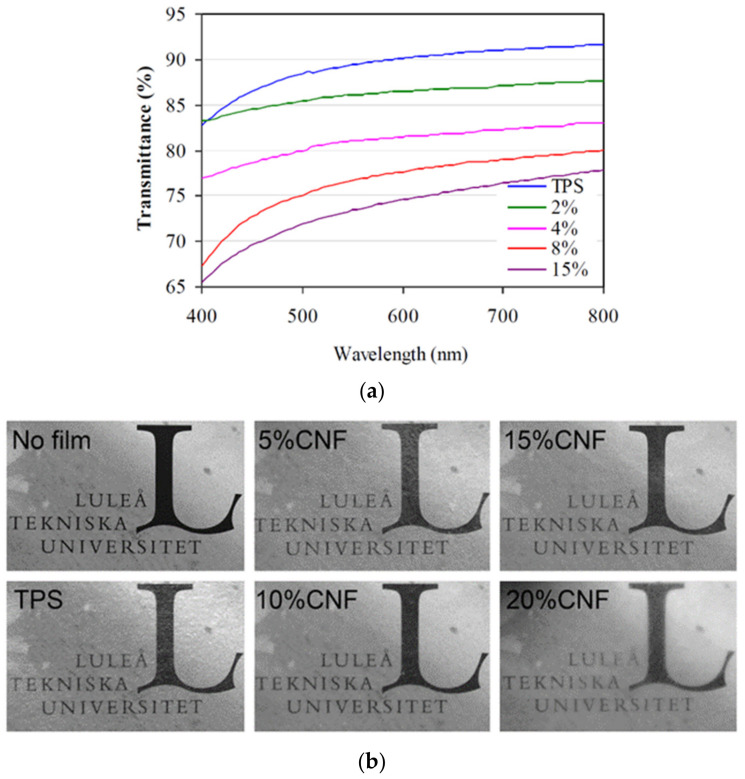
(**a**) UV-Vis transmittance spectra for TPS/CNF films. Reproduced with permission from *Carbohydr. Polym.*, 229, Fourati et al. One-Step Processing of Plasticized Starch/Cellulose Nanofibrils Nanocomposites via Twin-Screw Extrusion of Starch and Cellulose Fibers, 2020 [33]. (**b**) Visual aspect and transparency for TPS/CNF films at different wt %. Reproduced with permission from *Eur. Polym. J.*, *49*, Hietala et al., Bionanocomposites of thermoplastic starch and cellulose nanofibers manufactured using twin-screw extrusion, 950–956, 2013 [106].

The presence of CNF will affect the optical properties as well; this nanofiller in the TPS matrix could produce opacity in films as its content increases and depends on the quality of the dispersion of CNF in the matrix. Figure 15 shows the decrease in transmittance and clarity with the CNF addition; the authors agree that the poor dispersion of the CNF and the presence of agglomerates is the origin of the loss in the transparency [33,106].

**Figure 16:**
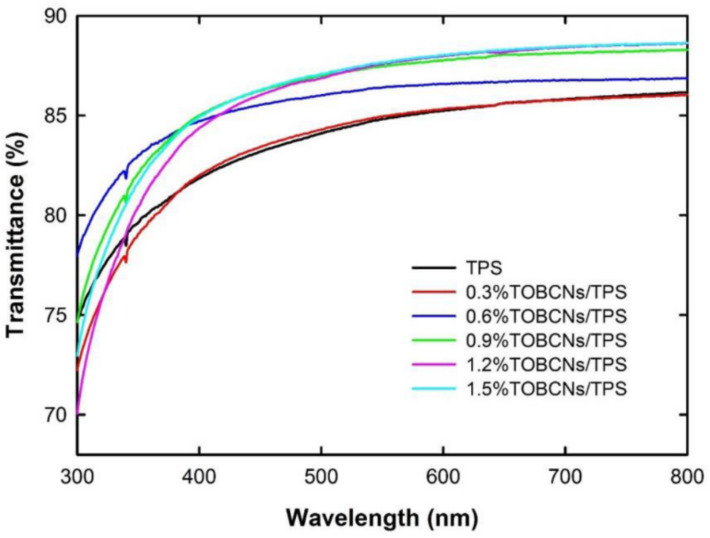
Light transmittance spectra of TOBCNs/TPS nanocomposite films. Reproduced with permission from *BioResources*, *14*, Pitiphatharaworachot et al., Starch nanocomposites reinforced with TEMPO-oxidized cellulose nanofibrils derived from bamboo holocellulose, 2784–2797, 2019 [81].

In some studies, the CNF improves the transparency of the composite. It is possible to increase the dispersion by improving the blending mechanism or applying chemical modifications. For example, Pitiphatharaworachot et al. [81] studied the TEMPO-oxidized (TEMPO stands for 2,2,6,6-tetramethylpiperidine-1-oxyl radical) bamboo cellulose nanofibrils (TOBCNFs) in tapioca TPS matrix (starch: glycerol 4:1). All films with the different nanofiller contents were homogeneous and transparent, owing to the good dispersion and bonding with the TPS matrix, increasing transparency by 3% at 600 nm (see Figure 16). This improvement is due to the nanometric size of TOBCNFs (3–4 nm of diameter), which is lower than optical wavelengths.

Another important characteristic is the degradability of the nanocomposite once the CNF is added. According to the reported studies, these nanocomposites are biodegradable. However, adding a highly crystalline phase to the TPS leads to a slight decrease in the biodegradation rate. This behavior was reported by Babaee et al. [117], who studied corn TPS nanocomposites with modified kenaf bast CNF. They degraded the samples using white-rot fungus, which consists of a laboratory incubator with purified fungi at 25 °C. These microorganisms are placed in a Petri dish until they are completely spread on the medium; then, the samples are placed on the medium on a platform to avoid direct contact. They are taken to an incubator at room temperature and 75% of relative humidity during the study time with periodic monitoring, recording the final weight after exposure.

**Figure 17:**
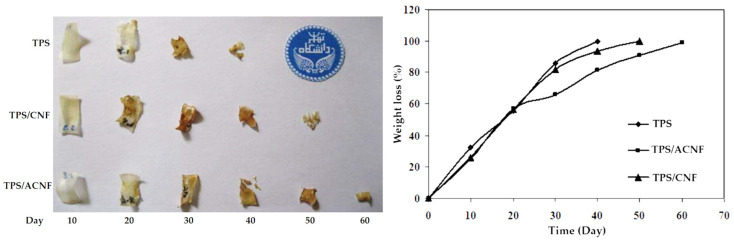
Visual and graphical fungal degradation of TPS and its nanocomposites. Reproduced with permission from *Carbohydr. Polym.*, *132*, Babee et al., Biodegradability and mechanical properties of reinforced starch nanocomposites using cellulose nanofibers, 1–8, 2015 [117].

Figure 17 shows the weight loss over time for TPS and nanocomposites with acetylated CNF and sole CNF. The compactness of the films is reduced after exposure by 50% within the 20 first days. Complete fungal degradability is different for the cases shown: for TPS, it is day 30, with CNF, it is at day 40, and acetylated CNF is day 60. CNF increases the crystallinity of the material, thus giving more resistance to degradation. These additions can restrict the destructive enzymatic activity and enzymatic hydrolysis of cellulose, increasing the degradation time compared to net TPS [117].

## 5. Cellulose Nanocrystals (CNC)

Similar to CNF, CNC can be obtained from a wide variety of natural sources. Table 2 shows the different starch and CNC sources extracted from several raw materials. These can be produced by a two-step mechanism that begins with acid hydrolysis, commonly HCl or H_2_SO_4_. This treatment is done to separate the amorphous region of the cellulose polymer, stabilizing the CNC in solution and preventing agglomeration. As the second step, and to obtain CNC, mechanical stress is required [76]. Some of the main key advantages of CNC include its easy combustion recyclability, low manufacturing energy, high availability, low density, non-abrasive nature (easy processability), and low cost [85].

From Table 2, we can deduce that the content of CNC on the TPS-based nanocomposites is similar to the employed in CNF, between 1 and 30%. The primary purpose is to increase the mechanical strength and elastic modulus. Achieving the desired properties requires homogeneous dispersion and strong hydrogen bonding between the filler and matrix molecules (reinforcement effect). Similarly, the decrease in elongation is present because of the strong interactions that reduce mobility between nanocrystals and the TPS matrix. On the other hand, a lack of interaction between the matrix and the fillers causes weak force transmission with rapid rupture propagation. A good balance is obtained by Cao et al. [79], who studied hemp cellulose nanocrystals (acid-catalyzed hydrolysis) in pea starch with glycerol matrix. They found in a range from 0 to 30 wt % of nanofiller values of 3.9 to 11.5 MPa for tensile strength, 31.9 to 823.9 MPa for modulus, and 68.2 to 7.5% for elongation at break.

**Figure 18:**
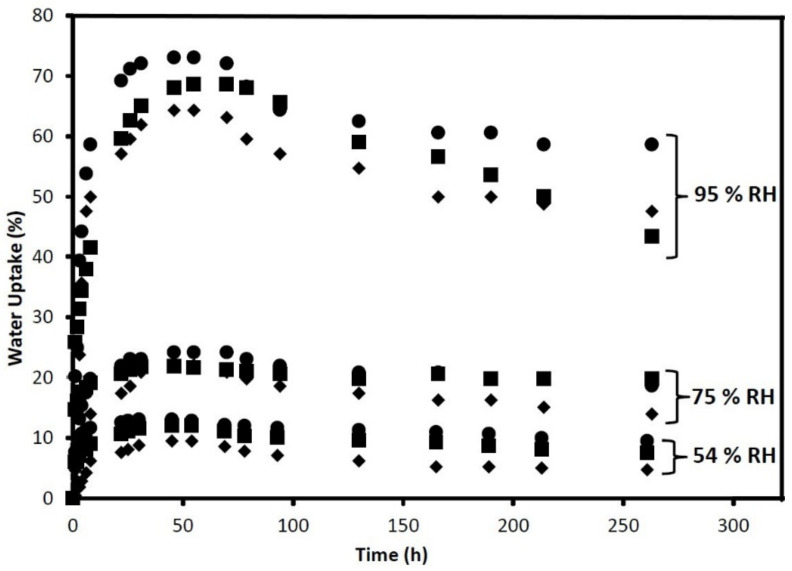
Water uptake trend through time considering the relative humidity (RH) at 20 °C for potato, pea, and corn TPS with CNC 5 wt %. Reproduced with permission from *Carbohydr. Polym.*, *157*, Montero et al., Effect of Nanocellulose as a Filler on Biodegradable Thermoplastic Starch Films from Tuber, Cereal, and Legume, 1094–1104, 2017 [5].

The mechanical properties and the overall performance of the nanocomposites are affected by the water uptake, since it facilitates the retrogradation process due to the increased molecular motion of starch molecules. It is necessary to determine the relative humidity (RH) at room temperature to evaluate the absorption capacity of the film. Figure 18 shows the water uptake trends at different values of RH for potato, pea, and corn TPS with glycerol 30 wt % and CNC at 5 wt % studied by Montero et al. [5]. This work was done by placing the films in different RH chambers (20 °C) at 95%, 75%, and 54%. Data are reported within 300 h, showing that the absorption rate is the same regardless of the starch source. Water is absorbed in the beginning of exposure until equilibrium is reached; then, retrogradation occurs, and water is released (turning material opaque, yellowish, and softer). It is shown that the filler content helped reduce the water diffusion through the material owing to hydrogen bonds between the filler and the matrix as well as increasing the crystallinity of the composite [5].

The addition of CNC potentially modifies the optical properties of TPS in terms of optical transparency, which is due to its size (nanofiller width); since it is smaller than the visible light wavelength, CNC allows the transmission of light in the matrix, making it a more transparent material [129]. The high transparency is given by the width of the nanocrystal and the adequate distribution and dispersion of the nanofiller in the matrix [130]. The effect is similar to that observed with a proper CNF incorporation.

As expected, the TPS–CNC nanocomposites are biodegradable, and their degradability is related to the process of depolymerization of nanocomposite by water and the hydrophilic character of CNC. The study mentioned above establishes that CNC increases the crystallinity of the material, and consequently, the hydrophobicity [5]. In contrast, Vaezi et al. [129] mentioned that CNC has a double effect: the first is the one indicated above, and the second is the increase in the rates of disintegration caused by the hydrophilic nature of the nanofiller after a period time of exposure to the environment. This evidence suggests that the degradation process started earlier in the nanocomposite than in net TPS.

**Figure 19:**
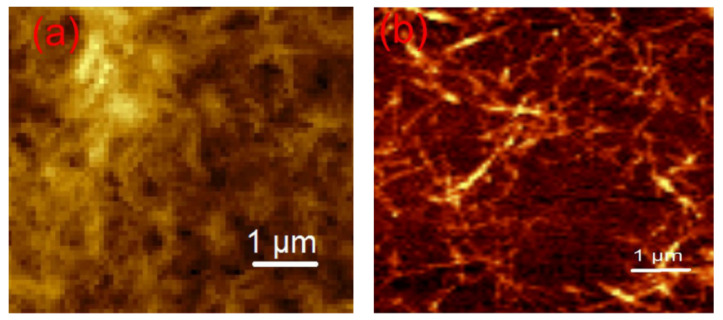
AFM of cellulose: (**a**) nanocrystals (CNC), (**b**) nanofibers (CNF). Reproduced with permission from *Macromol. Symp.*, *380* (*1*), Balakrishnan et al., Cellulose Nanofiber vs. Nanocrystals From Pineapple Leaf Fiber: A Comparative Studies on Reinforcing Efficiency on Starch Nanocomposites, 1–7, 2018 [37].

Comparing the effect of adding CNC or CNF to TPS, it is possible to find some differences; for example, the level of mechanical reinforcement is higher when the CNF is added; however, transparency tends to be reduced. The main reason for the difference is the nanofiller morphology. Figure 19 shows a short structure for CNC, while the suspension of CNF exhibits a higher L/D ratio. In addition, the crystallinity of the nanofiller is another critical factor; CNC has a higher crystallinity degree than CNF due to the elimination of amorphous regions in cellulosic arrays [37].

## 6. Natural Montmorillonite (MMT)

As a result of the TPS weaknesses, such as its hydrophilic nature, rapid degradation, and low performance, nanosized clays are a suitable option considering their properties. MMT shows an adequate distribution in the TPS matrix thanks to intercalation or exfoliation (Figure 8) [109]. Table 3 shows the different starch sources of TPS additivated with MMT that have been studied.

To prepare thermoplastic starch with nanoclays such as MMT, it is necessary to carry out a previous gelatinization process. Therefore, a starch/glycerol/nanoclay suspension is taken into an oven before the internal mixing process. This step is carried out to facilitate plasticization and optimize production by reducing process energy use during internal mixing [110].

The most noteworthy addition of MMT can be seen by the mechanical properties in the final product. An increase in elastic modulus and strength is observed as the nanofiller increases; this could be associated with the degree of exfoliation of MMT. Therefore, compatibility and dispersion with the TPS matrix are higher [109]. MMTs can withstand the effect of physical crosslinking with TPS and reinforcing it due to their surface area [131]; MMT decreases the elongation value, causing the applications of the material obtained to be restricted [132]. The main contribution of MMT is to the crystalline region (reducing the polymer chains’ mobility), thanks to the nucleation effect of the layers [133], but it does not affect the flexibility, which makes it ideal for their use in packaging [109].

**Figure 20:**
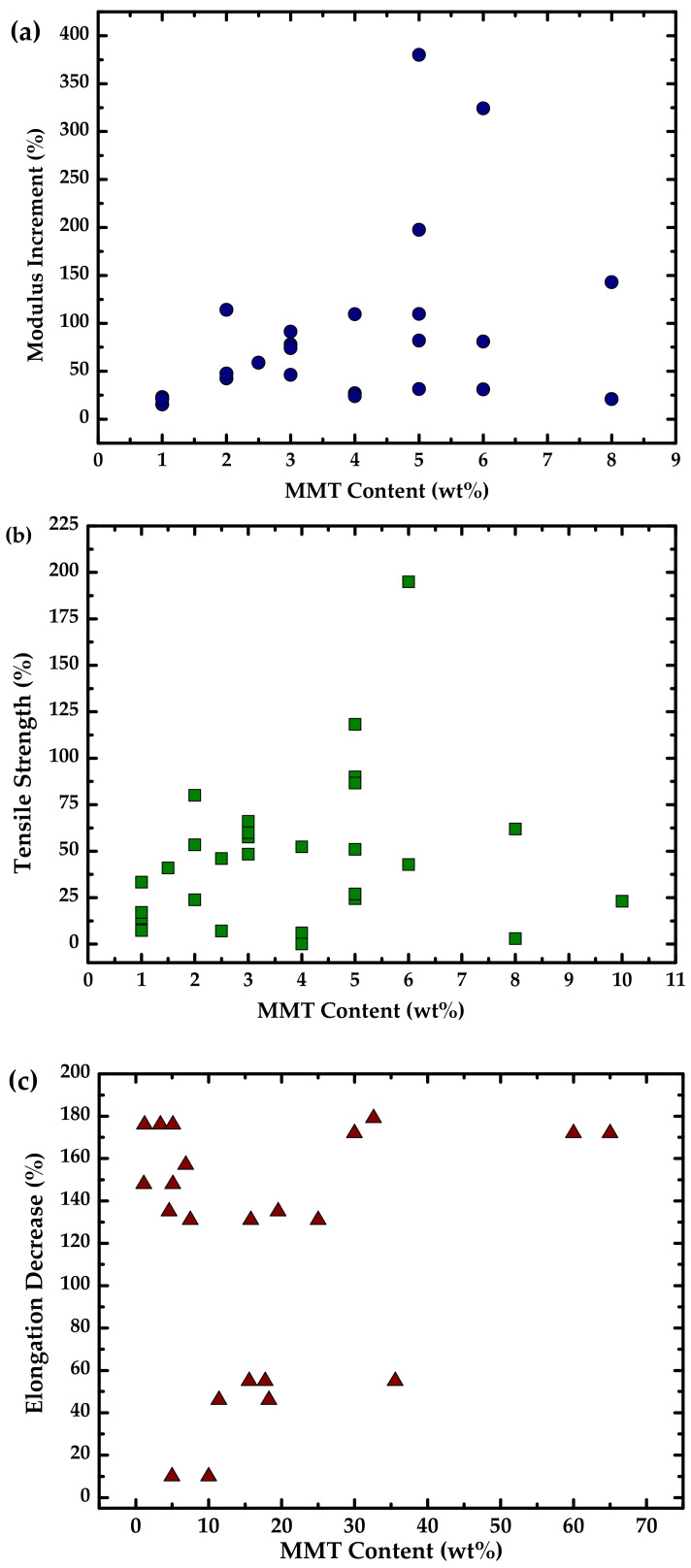
Mechanical properties with respect to montmorillonite (MMT) content: (**a**) tensile strength, (**b**) modulus increment, and (**c**) elongation decrease. Prepared from data in [43,44,49,53,63,83,100,109,131,136,143,154,155,157].

Figure 20 represents the elastic modulus, strength, and elongation to break concerning the MMT content, which is the most used nanofiller in the TPS matrix due to the high performance obtained. In the case of elastic modulus and strength (Figure 20a,b, respectively), they increase with the MMT content, and the dispersion cloud is higher between 2 and 6 wt % of MMT. However, the decrease in elongation (Figure 20c) does not follow a trend in its scattering. The behavior in the range (2 to 6 wt %) is due to the dispersion and distribution of the filler being adequate to improve the properties of the polymer. When the content is higher, agglomerates can be produced, decreasing the mechanical properties by reducing the stress distribution and increasing the rigidity. As the nanofiller content decreases, no significant change in the matrix is observed.

Ma et al. [131] studied corn TPS/MMT (2–10 wt %) nanocomposites (plasticized by sorbitol 0–20 wt %). They found the expected behavior for these nanocomposites. The Young modulus increased from 19.8 to 84.0 MPa, the tensile strength tripled to 12.27 MPa, and elongation decreased from 138.0% to 93.0%. This trend could be due to the adequate interaction between the matrix and the filler, which was intercalated (Figure 8b) and presenting a nano-scale dispersion. In addition, MMT presents physical crosslinking and TPS reinforcement, absorbing starch molecules thanks to its extensive specific surface. As mentioned above, it can be seen that the addition improves the elastic modulus and strength of the nanofiller. In this way, the mechanical properties are increased.

**Figure 21:**
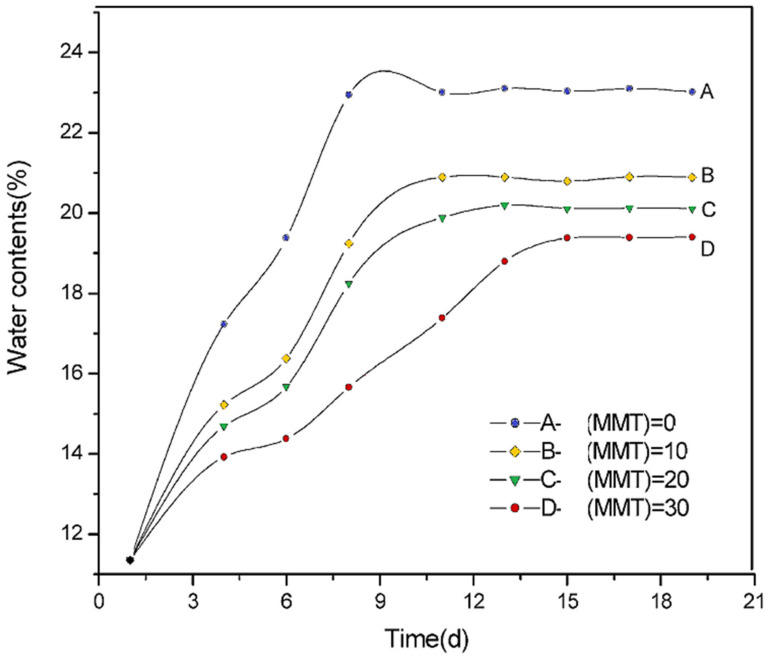
Water content of nanocomposites at 50% RH for different MMT in wt % (0, 10, 20, 30). Reproduced with permission from *Polymer* (*Guildf*)., *45*, Huang et al., Studies on the properties of montmorillonite-reinforced thermoplastic starch composites, 7017–7023, 2004 [158].

In order to consider different nanocomposite applications, water absorption is one of the main parameters in barrier properties to take into account. Huang et al. [158] studied corn starch/glycerol (1:3, *w*/*w*) with MMT content from 0 to 30% by extrusion. Figure 21 shows the water content through exposure to 50% RH environment for nanocomposites with MMT. It is observed that the rate of absorption gradually increases until reaching equilibrium around days 12 to 15 after exposure. A higher MMT content results in lower water uptake; this is described as stronger hydrogen bonds between the polymeric matrix and the nanofiller. This result can also be related to the mechanical properties: in a range from 5 to 50% of water content, the stress first increases (with a maximum at 13% of water content) and then decreases rapidly. It suggests that with the lowest and highest water content, the poor mechanical performance of the material will be exhibited.

In terms of optical properties, incorporating MMT in the TPS matrix decreases the luminosity and transparency due to the increase in light dispersion and diffuse reflectance, resulting in an opaquer nanocomposite. This behavior is highlighted by using rosemary essential oil (as an antioxidant) studied by Azevedo et al. [43] in corn TPS. It occurs due to a possible light scattering in the interface of oil droplets.

Additionally, ZnO nanoparticles along with MMT were studied by Vaezi et al. [44] in cationic starch (with glycerol) prepared by the solvent casting method. Although these nanoparticles reduce luminosity, the opacity is enhanced by the concentration of ZnO particles because it is considered a whitening agent. They also accentuated the difference in the color of the materials. TPS/MMT films additivated with ZnO particles can be UV-shielding and thermal insulators in the packaging industry.

**Figure 22:**
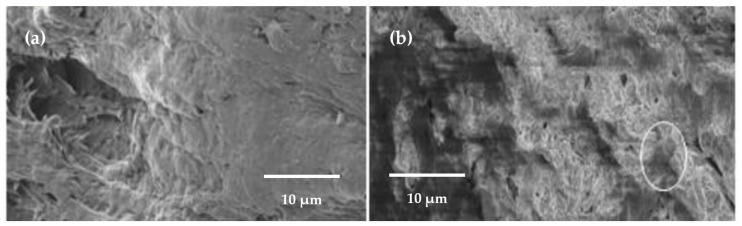
FE-SEM micrograph for TPS nanocomposite with 3 wt % of MMT (**a**) before degradation and (**b**) after degradation (60 days). Reproduced with permission from *IOP Conf. Ser. Mater. Sci. Eng.*, *410*, Behera, Mechanical and biodegradation analysis of thermoplastic starch reinforced nano-biocomposites, 2018 [159].

For the degradability study, Behera [159] prepared corn TPS with MMT from 0 to 5 wt % by extrusion. According to standard procedures, the biodegradation analysis was performed using the soil-burial method, and the results were studied by field-emission (FE) SEM (Figure 22). It is shown that after 60 days of burial, the weight loss of net TPS to TPS nanocomposite, with 3 wt % of MMT, is 9% greater for the latter. This improvement is attributed to microorganism attack burial in the first place (circled cavity in Figure 22b). Furthermore, it is evident that before degradation, the surface is smoother (Figure 22a) than after it. Since it is highly biodegradable, the potential replacement of conventional plastics is foreseen.

## 7. Organically Modified Montmorillonite (O-MMT)

To increase the compatibility of the nanofiller with the matrix, MMT is organically modified to form intercalated or exfoliated structures with suitable interlayer distances [131]. Table 4 shows different sources of starch with O-MMT with various treatments. An example of organic treatments includes the modification of MMT by Ren et al. [53] via an activation method with dodecyl benzyl dimethyl ammonium bromide (12-OREC). The O-MMT solution is heated slowly to 50 °C; then, 12-OREC is added, and this solution is brought to 90 °C for 5 h. Finally, the solution is cooled to room temperature, filtered, dried, and pulverized. Even though there are many variations of O-MMT, Cloisite 30B is the most commercially available and used.

**Figure 23:**
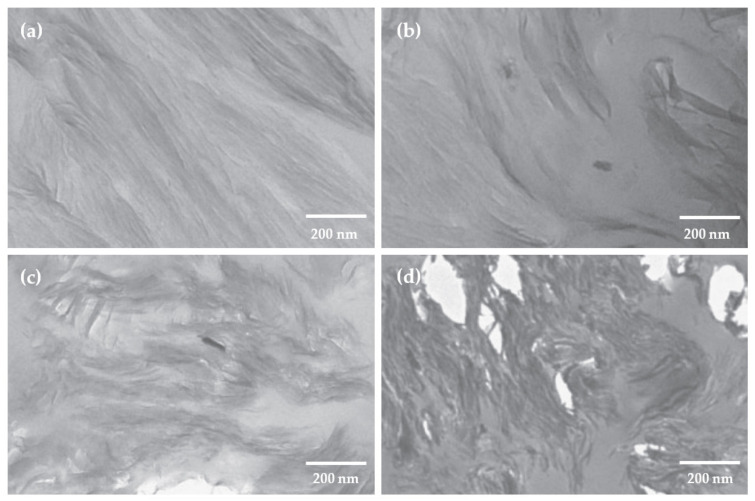
TEM micrographs of TPS matrix with different nanoclay concentrations in wt %: (**a**) 1, (**b**) 2, (**c**) 3, (**d**) 5. Reproduced with permission from *J. Plast. Film Sheeting*, *32*, Mohan and Kanny, Thermoforming studies of corn starch-derived biopolymer film filled with nanoclays, 163–188, 2016 [48].

Adequate mechanical performance of the O-MMT nanocomposite requires proper dispersion and distribution through the matrix. The general tendency to use low concentrations (up to 3 wt %) follows that the properties are optimized, while higher concentrations tend to form agglomerates in the matrix. For example, Mohan and Kanny [48] studied corn TPS with Cloisite 30B; when 1 and 2 wt % of nanoclay is present in the matrix, a uniform and exfoliated structure can be observed because of the well-separated and randomly dispersed filler. For higher contents, meaning 3 and 5 wt %, the level of agglomeration of the nanolayers is elevated, which leads to an intercalated nanocomposite structure (Figure 23).

The barrier and mechanical properties are better for exfoliated than intercalated arrangements (8). This improvement may be due to the aspect ratio (length/thickness); more contact surface of the clay with the matrix is shown in an exfoliated structure, reducing the net thickness and improving the dispersion. On the other hand, intercalated configurations exhibit a particular orientation of the clay layers, increasing net thickness and reducing the area of the contact surface of the filler with the matrix [48].

**Figure 24:**
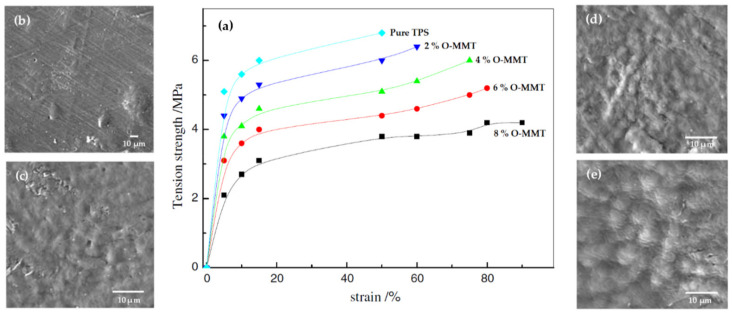
(**a**) Tension strength versus strain for neat TPS and the TPS/O-MMT nanocomposites indicated. SEM images of (**b**) 2% O-MMT, (**c**) 4% O-MMT, (**d**) 6% O-MMT, (**e**) 8% O-MMT. Reproduced with permission from *Polym. Environ.*, *17*, Ren et al., Study on biodegradable starch/OMMT nanocomposites for packaging applications, 203–207, 2009 [53].

The influence of the nanoclay configuration within the TPS matrix is evidenced by the work of Ren et al. [53]; they studied the effect of different content of modified MMT on the mechanical behavior and crystallinity of the TPS, finding an increase in the restriction of the granular, crystalline structure of starch in the nanocomposite with the rise of O-MMT, as can be seen in Figure 24. When the filler content increases in a moderate range, the elastic modulus and strength tend to improve, but elongation decreases with filler content. Although the TPS morphology is modified by the action of the filler, the low clay contents produce excellent dispersion in the TPS matrix, but high clay contents lead to agglomeration.

The agglomeration and nanoclay configuration affect the barrier properties as well. Boonprasith et al. [135] compared MMT and Cloisite 30B (5 pph) in TPS with poly(butylene succinate) (PBS) matrix (75/25% *w*/*w*) plasticized with glycerol (30 wt %). They found that WVP could not be measured because TPS is the major component in matrix, and it is sensibly hydrophilic. In terms of oxygen permeability, changes are not significant regardless of the clay type.

**Figure 25:**
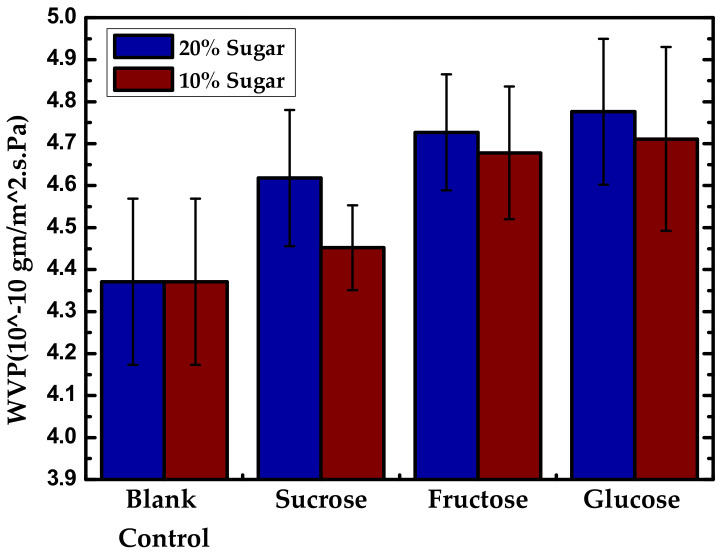
WVP of starch-based nanocomposite films with 20% and 10% sugar content at 6 wt % of modified MMT. Reproduced with permission from *Int. J. Biol. Macromol.*, *133*, Gao et al., The Co-Plasticization Effects of Glycerol and Small Molecular Sugars on Starch-Based Nanocomposite Films Prepared by Extrusion Blowing, 1175–1181, 2019 [165].

Gao et al. [165] studied films made by extrusion blown with hydroxypropyl starch, glycerol, O-MMT, and sugars (10 or 20%) as co-plasticizing agents (sucrose, fructose, and glucose). Figure 25 shows an increase in WVP with sugars due to their hydrophilic nature and molecular expansion effect in the plasticization phase.

In the case of optical properties and biodegradability of TPS/O-MMT nanocomposites, more research should be conducted because there is little information in the literature, mainly studying these nanofillers in other matrixes. However, Mohan and Kanny [48] point out that Cloisite 30B increases the degradation rate in the soil burial test when compared with MMT-based TPS and net TPS.

## 8. Other Nanofillers

In addition to the main nanofillers mentioned above, studies were conducted with various nanofillers and starch sources, as can be seen in Table 5. In this section, some examples regarding their mechanical properties and degradability will be briefly presented, which are the main parameters to consider for TPS nanocomposites analysis and end-product applications.

As shown in Table 5, the TPS has been blended with other natural nanofillers such as chitosan, bacterial cellulose, bentonite, kaolinite, and synthetic ones such as carbon nanotubes, silver, and graphene quantum dots. In most cases, the amount employed was low, around 5 wt %.

In all the studies, the mechanical properties were affected. For example, the tensile and flexural properties are enhanced by multi-walled carbon nanotubes (MWCNTs). In general, they are acid-functionalized with a mixture of sulfuric and nitric acids [2] to improve their merging into the matrix. This chemical modification increases the filler hydrophilicity and reduces the agglomerations in the matrix, which contributes to hydrogen bonding interactions and compatibility. Cao et al. [118] studied this filler in pea starch plasticized with glycerol and water. They obtained in a range from 0 to 3 wt % of nanofiller, 2.85 to 4.73 MPa for tensile strength, 20.74 to 39.18 MPa for elastic modulus, 41.99% (maximum) at 1 wt % of filler for elongation at break (if the filler exceeds 1 wt %, elongation slightly decreases).

Although the general increasing trend mentioned in the other system for elastic modulus and strength and a decreasing trend for elongation is maintained in most studies, it sometimes does not follow that rule. That is the case of the work of Kwaśniewska et al. [181], showing that in the nanocomposite with 15% kaolinite in potato TPS matrix (with 20 wt % of glycerol), the modulus and strength is lower than the base film, and the elongation increases. These changes could be due to the intercalation of kaolinite in the TPS matrix.

**Figure 26:**
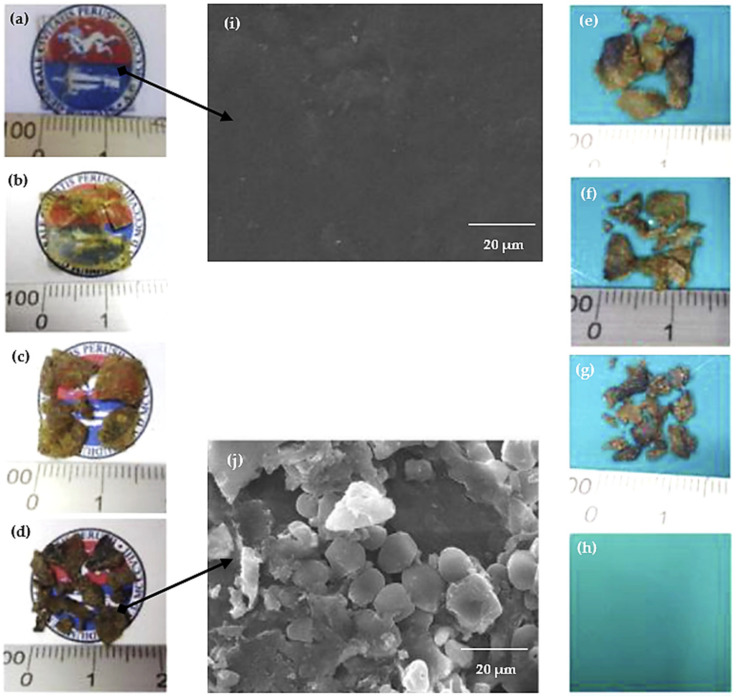
Disintegration process under composting conditions of TPS film at day (**a**) 0, (**b**) 1, (**c**) 11, (**d**) 18, (**e**) 25, (**f**) 32, (**g**) 39, and (**h**) 56. SEM micrograph of the disintegration process at day (**i**) 0 and (**j**) 18. Reproduced with permission from *Polym. Degrad. Stab.*, *159*, Sessini et al. Thermal and composting degradation of EVA/Thermoplastic starch blends and their nanocomposites, 184–198, 2019 [45].

As was discussed for the other nanofillers, the biodegradation of these nanocomposites is worth mentioning. Its behavior should depend on the nature and amount of the nanofiller. However, there is not enough data to establish a general trend. One example of the TPS-based biodegradation behavior is the study of Sessini et al. [45], who prepared EVA/pea TPS (50:50) blend nanocomposites with glycerol (25 wt %) and distilled water (20 wt %) reinforced with natural bentonite (1 wt %). Figure 26 shows the visual disintegration process of TPS under aerobic conditions until its complete disappearance at day 56. A yellowish tone and breakable structure are reached even on day 1 (Figure 26a), which is caused by the enzymatic attacks of microorganisms that break the bonds of long-chain sugar units. In addition, the opaque tone is caused by the hydrolytic disintegration process. The environmental impact can be improved because these materials take less time to degrade, considering that composting conditions would be required.

Different arrangements can be made as test options for degradability studies. One of the most common is to bury the material in a mixture that reproduces outdoor aerobic conditions of the environment. For example, composting conditions may include a solid synthetic waste made of 10% compost, 30% rabbit food, 10% starch, 5% sugar, 4% corn oil, 1% urea, and 40% sawdust with a humidity content of 50%. The material is cut into small samples and placed on a textile to perform the disintegrability test under these parameters. This arrangement allows for quick removal after the test and easy access to moisture and microorganisms. The synthetic residue is buried 4 to 6 cm deep, and the synthetic waste is incubated under aerobic conditions (58 °C). Weight normalization is necessary to measure disintegrability, which is relative to the initial day after the cleaning and drying [45].

## 9. Analysis and Discussion for Packaging Purposes

It seems impossible to think of life without plastic, but it is one of the major pollutants in many aspects of daily life and industries. Currently, landfills are the most widely applied method to reduce packaging waste disposal; however, more methods include incineration, recycling, and compost. According to American Society for Testing and Materials (ASTM), biodegradable and compostable are not equivalent terms. Biodegradation considers the environment (temperature, moisture, oxygen, pH) in which the material is placed and the chemical nature of the polymer, while composting refers to a material that suffers degradation by microorganisms to produce biomass, water, carbon dioxide, and inorganic compounds at a consistent rate. All compostable materials are biodegradable, but the opposite does not come into effect [183].

Bio-based polymers have been extensively developed with new technologies to address this problem, reducing costs and improving performance. In addition, studies suggest that multi-layer arrangements or blends accomplish better performance for bio-based materials. In order to meet the standards for synthetic polymers, requirements include adequate water vapor permeability, resistance to water, acids, oils, UV light, machinability, transparency, and anti-fogging capacity availability, among others [184].

TPS nanocomposites reinforced with CNF, CNC, MMT, and O-MMT will be analyzed for this application regarding the items presented above and considering the main factors for ideal packaging material. TPS nanocomposites are an excellent option for packaging thanks to their high availability, easy processability, biodegradability, and compostability; however, the addition of fillers enhanced these properties [12].

TPS/CNF nanocomposites may be suitable for packaging material because they meet the requirements mentioned above. Adequate plasticizer and proper dispersion, distribution, and interaction with the matrix lead to favored mechanical and barrier properties, using on average 15 wt % [33]. However, if aesthetic characteristics are required for the end usage material, it is essential to know that an excessive CNF (around 5–20 wt %) confers opacity [106], while low content (less than 1.5 wt %) results in increased transparency [81]. The degradation rate will be related to a modification in CNF; e.g., if the material will be needed for prolonged use, the acetylated CNF confers a longer degradation time after exposure than sole CNF (short-term use) [117].

Crystallinity is the main factor in CNC because, unlike CNF, its incorporation directly affects the crystalline region of TPS [37]. The stiffness of the material will increase, causing a more pronounced reduction in elongation due to reduced mobility of starch molecules, and it will maintain strength and the modulus capacity under competent conditions [79]. Improvements in barrier properties are observed when the material begins to be exposed to the environment [5], but the hydrophilic nature of CNC produces earlier degradability than the rest of the TPS matrix after a specific exposure time [129]. Considering that CNC is smaller than the optical wavelength, they are useful for packaging applications when the esthetic factor is the most determinant in the final product (they improve material transparency) [130].

Likewise, the increase in CNC and MMT crystallinity in TPS materials does not drastically affect flexibility, which improves mechanical properties (around 2–10 wt %) [109,131]. For this reason, field packaging is notably considered among their applications. Furthermore, the strong interaction between the nanofiller and the matrix leads to adjusting barrier properties in a relative range of 0 to 30 wt %. The chosen MMT content is crucial for the WVP and, as a consequence, for the mechanical performance of the nanocomposites [158]. On the other hand, the appearance of the material will be affected by this nanofiller because it causes an increment in opacity, so it has to be considered if transparency is of importance for the packaging [44]. These nanocomposites fulfill the characteristics of biodegradable packaging, as was shown by the soil-burial tests that have been carried out [159].

In TPS/O-MMT, these nanocomposites can be applied in the packaging industry, specially reinforced with Cloisite 30B because of the high commercial availability (organic modification is already done), reducing processing costs. In addition, the mechanical and barrier properties are optimized thanks to a good interaction with the matrix, forming exfoliated structures. The inclusion of O-MMT produces a higher biodegradation rate, making it efficient for packaging use [48]. In order to analyze the esthetics of the final product, it is necessary to carry out more studies in optical properties.

## 10. Final Remarks and Future Perspectives

TPS is a suitable option to replace conventional thermoplastic polymers; however, the lack of mechanical properties exhibited by TPS alone can be overcome by adding nanofillers. This review focused on the following four common ones: MMTs (natural and organically modified), CNC, and CNF. These fillers must have a good dispersion and distribution in the TPS matrix, which leads to better mechanical properties (increased modulus and strength while decreasing elongation) and barrier properties (greater hydrophobic character). On the other hand, the optical properties (transparency and luminosity) are mostly reduced, and the color variation with these fillers needs further study. In the case of biodegradability, few studies have been carried out with the soil-burial method.

Despite that, more research of new methods to reproduce different conditions is needed or even implementing the material in real scenarios to obtain more information on the degradation rate. The study of different starch blends can be useful to redirect the potential of TPS usages in terms of properties and morphology. Other unconventional sources for starch and different type of fillers could be explored further. The interaction of the polymer with nanofiller is another potential prospect for considerable studies in order to reduce deformation at break. This improvement could be made by chemical modification of either the TPS or the nanonanofiller. Due to its good performance, TPS with nanofillers is a promising option for new advanced materials for the packaging industry. Finally, the processing methods for these nanocomposites and other similar systems should be systematically studied to establish their applicability in real life.

## Figures and Tables

**Table 1:** Plasticizers and cellulose nanofibers as nanofillers (including their wt %) in most used TPS sources.

Starch Source	Plasticizer	Nanofiller	Wt %	Reference
NI	Glycerol	Cotton nanofibers	0.1–1	[116]
Cassava	Glycerol/Sorbitol (1:1)	Cellulose cassava bagasse nanofibers	5–20	[70]
Corn	Glycerol	Cellulose nanofibers (eucalyptus pulp)	2–15	[33]
	Glycerol	Cellulose nanofibers	2–12	[35]
	Glycerol	Cellulose nanofibers	1–30	[36]
	Glycerol	Cellulose nanofibers	5	[12]
	Glycerol	Cellulose nanofibers	10	[117]
	Glycerol	Cotton cellulose nanofibers	0.5–5	[118]
	Glycerol	Graphene oxide nanoplatelets, Cellulose nanofibers	1–5, 5–15	[119]
	Glycerol	Lignin cellulose nanofibers	5–15	[120]
Corn, cassava, sago	Glycerol, formaldehyde	Oil palm empty fruit bunches cellulose nanofibers	1–3	[121]
Maize	Sorbitol	Sugarcane bagasse cellulose Nanofibers	4–20	[77]
	Glycerol and sorbitol	Cotton nanofibers	5–20	[122]
	Glycerol	Cellulose nanofibers	5–15	[123]
Merck-modified starch	Glycerol	Rice straw cellulose nanofibers	5–15	[124]
Potato	Glycerol	Cellulose nanofibers and nanocrystals	1–3	[37]
	Water/Glycerol	Sisal cellulose nanofibers	2.5–20	[62]
	Glycerol	Wheat straw cellulose nanofibers	2–10	[64]
	Glycerol	Pineapple leaf cellulose nanofibers	1–4	[52]
	D-Sorbitol	Cellulose nanofibers	5–20	[106]
	Glycerol	Bleached eucalyptus pulp cellulose nanofibers	0.18–0.36	[125]
Tapioca	Glycerol	Cellulose nanofibers	0.3–1.5	[81]
Waxy maize	Glycerol	Cellulosic nanofibers	2–10	[73]

NI: No information reported.

**Table 2:** Plasticizers and cellulose nanocrystals as nanofillers (including their wt %) in most used TPS sources.

Starch Source	Plasticizer	Nanofiller	Wt %	Reference
NI	Glycerol	Cellulose nanocrystals	5	[126]
Corn	Glycerol	Waxy corn starch nanocrystals Cellulose nanocrystals	1–5	[40]
	Glycerol	Cellulose nanocrystal	1–2	[127]
	Glycerol	Starch nanocrystals	1–2	[41]
Field pea	Glycerol, concentrated sulfuric acid, and sodium hypochlorite solution	Hemp cellulose nanocrystals	5–30	[79]
Maize	Glycerol	Waxy starch nanocrystals (WSNC)/Cellulose cellulose nanocrystals	1–5	[38]
	Glycerol	Cotton cellulose nanocrystals	4–8	[74]
	Glycerol	Cellulose nanocrystals	5–25	[75]
	Glycerol	Waxy maize starch nanocrystals	2.5	[42]
Potato	Glycerol	Cellulose nanocrystals	1.5–10	[34]
	Glycerol	Cellulose nanofibers and nanocrystals	1–3	[37]
	Glycerol	Cellulose nanocrystals	1–2	[128]
	Glycerol	Waxy maize nanocrystals	5–15	[39]
Potato, corn, pea	Glycerol	Cellulose nanocrystals	2–5	[5]

NI: No information reported.

**Table 3:** Plasticizers and montmorillonite as nanofiller (including their wt %) in most used TPS sources.

Starch Source	Plasticizer	Nanofiller	wt %	Reference
Acetylated cassava	Water	Montmorillonite	1–10	[69]
Cassava	Glycerol	Montmorillonite, alumina trihydrate	26–37, 1–6	[67]
	Glycerol	Montmorillonite	3–5	[133]
	Glycerol	Montmorillonite, Cloisite 30B	5	[68]
	Glycerol	Na-montmorillonite (Closite^®^ Na+)	1–2	[71]
	Glycerol	Montmorillonite	2–4	[134]
	Glycerol	Sodium montmorillonite, modified organo-montmorillonite	NI	[135]
Cationic starch	Glycerol	Sodium montmorillonite, ZnO	3–5, 0.5–1	[44]
Cereal	Glycerol	Montmorillonite/Chitosan	3–5/0.6–1	[49]
Corn	Glycerol	Sodium montmorillonite	1	[43]
	Glycerol/water	Montmorillonite clay	3–4.5	[86]
	Glycerol/water	Hydrophilic bentonite, sodium montmorillonite/Essential oils constituents	0.5	[47]
	Glycerol	Walnut shell flour/Montmorillonite (MMT)	30–50/3–5	[89]
	Glycerol	Montmorillonite	0–5	[83]
	Glycerol	Montmorillonite	1–6	[84]
	Glycerol	Sodium montmorillonite	3–5	[136]
	Glycerol	Montmorillonite	1–5	[137]
	Glycerol	Sodium montmorillonite	2–5	[138]
	Glycerol	Sodium montmorillonite	2–8	[138]
	Glycerol	Montmorillonite clay	1–5	[51]
	Glycerol/water	Montmorillonite clay	1–9	[139]
	Glycerol	Montmorillonite clay	1–5	[109]
	Water	Sodium montmorillonite clay	5	[140]
	Glycerol	Montmorillonite (natural and glycerol-activated)	1–9	[141]
	Glycerol	Natural montmorillonite	2–6	[97]
	Glycerol	Sodium montmorillonite	1–9	[142]
	Water, partially hydrolyzed polyvinyl alcohol	Natural montmorillonite	1–5	[143]
	Sorbitol, formamide	Sodium montmorillonite	2–10	[131]
	Water	Natural montmorillonite, fluorohectorite	1–3.2	[144]
	Citric acid, formamide, and ethanolamine	Sodium montmorillonite	2–10	[145]
	Glycerol	Montmorillonite	0.03–0.1 (g)	[146]
Corn, wheat, potato	Glycerol	Natural montmorillonite, Cloisite 30B	3–15	[147]
Granular Maize	Glycerol	Montmorillonite	1–7	[72]
Maize	Glycerol	Natural montmorillonite, Cloisite 30B	5	[148]
	Glycerol	Montorillonite	10–20	[149]
Merck starch	Glycerol	Natural montmorillonite	1–5	[150]
Pearl silver corn starch	Glycerol	Natural montmorillonite, Cloisite 30B	1, 1–5	[80]
Potato	Glycerol	Sodium montmorillonite	2–5	[63]
	Urea	Montmorillonite		[151]
	Glycerol	Montmorillonite, kaolinite, hectorite and treated hectorite	6–22/5–18/5–20/5–19	[152]
	Glycerol	Organically modified montmorillonite (Cloisite 30B), Natural montmorillonite Sodium montmorillonite	2.5–10	[153]
	Glycerol	Sodium montmorillonite	2–5	[63]
	Glycerol/water	Sodium montmorillonite	1–1.5	[154]
	Glycerol	Montmorillonite	4–8	[155]
	Glycerol/water	Cloisite 30B, Cloisite 10A, Cloisite 6A and Sodium montmorillonite	5	[102]
Sweet potato	Carbamide and ethanolamine	Sodium montmorillonite	2–8	[148]
Tapioca (Acetylated)	Glycerol	Natural and organically modified montmorillonite	5	[150]
Wheat	Glycerol	Sodium montmorillonite, Aminododecanoic-acid-treated organophilic clays	Silicate content: 0.5–7 (vol)	[80]
	Water/Glycerol	Montmorillonite	2–5	[87]
	Glycerol	Montmorillonite, Cloisite 30B, Cloisite 10A	1–5	[156]

Note: Natural montmorillonite and sodium montmorillonite refer to the same nanofiller. NI: No information reported.

**Table 4:** Plasticizers and organically modified montmorillonite as nanofiller (including their wt %) in most used TPS sources.

Starch Source	Plasticizer	Nanofiller	wt %	Reference
NI	Glycerol	Cloisite 30B	3	[160]
Cassava	Glycerol	Montmorillonite, Cloisite 30B	5	[68]
	Glycerol	Sodium montmorillonite, modified organo-montmorillonite	NI	[135]
Corn	Glycerol	Cloisite 30B	1–5	[48]
	Sorbitol	Closite 30B	1–5	[161]
	Glycerol	Montmorillonite clay	1–5	[51]
	Glycerol	Cloisite 30B	2.5–10	[162]
	Glycerol	Montmorillonite (natural and glycerol-activated)	1–9	[141]
	Glycerol	Pristine clay (p-clay), Cloisite 93A	3	[163]
Corn, wheat, potato	Glycerol	Natural montmorillonite, Cloisite 30B	3–15	[147]
Maize	Glycerol/distilled water	Bentonite and organically modified montmorillonite	40–50	[76]
	Glycerol	Natural montmorillonite, Cloisite 30B	5	[148]
Pearl silver corn starch	Glycerol	Natural montmorillonite, Cloisite 30B	1, 1–5	[80]
Potato	Glycerol/water	Cloisite (organoclay)	5	[164]
	Glycerol	Cloisite 30B, natural sodium montmorillonite	2.5–10	[100]
	Glycerol/water	Cloisite 30B, Cloisite 10A, Cloisite 6A and Sodium montmorillonite	5	[102]
Tapioca (Acetylated)	Glycerol	Natural and organically modified montmorillonite	5	[82]
Wheat	Glycerol	Sodium montmorillonite, aminododecanoic-acid-treated organophilic clays	Silicate content: 0.5–7 (vol)	[153]
	Glycerol	Montmorillonite, Cloisite 30B, Cloisite 10A	1–5	[156]

Note: Natural montmorillonite and sodium montmorillonite refers to the same nanofiller. NI: No information reported.

**Table 5:** Plasticizers and other nanofillers (including their wt %) in most used TPS sources.

Starch Source	Plasticizer	Nanofiller	wt %	Reference
NI	Glycerol	Chitin nanofibers	3–10	[166]
	Glycerol	Silver nanoparticles	0.5–1	[90]
Cassava	Glycerol	Chitosan-modified Veegum^®^ HS clay (smectite)	2.5–5	[65]
	Glycerol	Sepiolite	1–5	[66]
	Glycerol	Sepiolite	1–5	[167]
	Glycerol	Silver nanoparticles	0.006–0.15	[168]
	Glycerol	Halloysite nanotubes	2	[169]
	Glycerol	Halloysite nanoclay	1–5	[170]
	Glycerol	Halloysite nanotubes	2–8	[171]
Corn	Glycerol	Talc nanoparticles	1–5	[87]
	Glycerol	Graphene quantum dots (GQD)	0.05–0.5	[88]
	Glycerol	Laponite	1–5	[172]
	Glycerol	Carboxylate multi-walled carbon nanotubes (CMWNTs)	0.5–3	[173]
	Glycerol	Bentonite, chitosan	4	[174]
	Glycerol	Talc nanoparticles	0–5	[175]
	Glycerol	Bacterial cellulose nanowhiskers (BCNW)	2–20	[50]
	Glycerol	Talc	1–5	[176]
	Glycerol	Bentonite, organically modified montmorillonite	40–50	[76]
	Glycerol	Beta-tricalcium phosphate nanoparticles	3–10	[177]
	Glycerol	Nanoclay: bentonite (H_2_Al_2_O_6_Si)	1–5	[178]
	Sorbitol	Cardanol oil, in situ silver nanoparticles	0.2–0.6, 1–4 (mmol)	[179]
	Glycerol	Graphene oxide nanoplatelets, cellulose nanofibers	1–5, 5–15	[119]
Maize	Glycerol	Lanthanum hydroxide nanoparticles	1–3	[180]
	Ethyl vinyl acetate	Bentonite		[45]
	Glycerol	Zirconium glycine-N,N-dimethylphosphonate (ZGDMP)	0.2–1	[77]
Pea	Glycerol	Acid-treated multi-walled carbon nanotubes (MWCNTs)	0.1–3	[80]
	Glycerol	Natural bentonite	1	[78]
	Polyethylene glycol and glycerol	Particles of AgNO_3_, Silver	2.5–5, 0.5–1	[90]
Pomegranate	Glycerol	Halloysite nanoclay	3–7	[46]
	Glycerol	Talc, bentonite	1–5, 1–5	[59]
Potato	Glycerol	Bacterial cellulose (BC) nanoribbons		[60]
	Glycerol	Kaolin clay	5–15	[181]
	Glycerol	Multi-walled carbon nanotubes (MWCNT)	0.25–10	[2]
Tapioca	Glycerol	Kaolinite	10–60	[182]
	Glycerol, urea, ethanolamine	Halloysites nanotubes	6	[54]

NI: No information reported.
